# Tattooing

**Published:** 1873-01

**Authors:** 


### TATTOOING.

It is with considerable pleasure that we are enabled to announce that tattooing, that fanciful discoloration of the skin which is not entirely confined to savages, but has numerous patrons among our sailors and school boys, has at last been found to be of real service to mankind.

We have all seen the blue and red marks indelibly wrought upon the skin, representing various and multitudinous devices, according to the fancy of the thoughtless school-boy who was foolish enough to imprint them upon his arm, and who would give fabulous sums to have them as successfully removed, when he has reached the age of discretion. Well, as we have intimated, the process has just been discovered to be of actual and wonderful service in certain deformities of the eye. In opacities of the cornea,—that terrible disfigurement to the features, in which the eye presents that glaring white appearance, the result of ulceration of the cornea, tattooing is resorted to, by means of which the white spot can be colored in exact imitation of the sound eye, so that the blindness is not apparent to any one but the patient. If the white spot is large, covering the entire cornea, a round, perfectly black spot is picked in the centre of the cornea, in imitation of the pupil, while the balance is filled in with colored pigment tinted to imitate the natural iris, as blue, brown, etc. Coloring materials of various hues can be used, as water colors, and india-ink, the only requisite being that they shall be unirritating and insoluble.

It is not only for its cosmetic effects that tattooing has been found to be valuable, but is also used to the manifest improvement of vision, in several optical defects in the eye. It has been successfully used for shutting out the excessive light from the eyes of albinos, who, our readers are aware, are unable to see distinctly by reason of a lack of proper pigmentary deposit in the iris, which defect allows such a flood of light to enter the eye, as to render vision almost useless. By tattooing the cornea with black or dark pigment, this defect is remedied.

In congenital absence of the iris, or where that membrane is partially or wholly destroyed by accident, a very good substitute is found in tattooing. Astigmatism, conical cornea, and other defects of the cornea are greatly relieved through the same means.

The process is easily accomplished, and is attended by very little, and in some instances no pain at all. The pigment of the desired color, is made in the form of a paste, spread upon the cornea, and then pricked into its layers by means of a little instrument, suited to the purpose. The instrument ordinarily used is made by binding several cambric needles about a pen-holder, or other round stick, with which the operation is quickly done, by going rapidly over the surface of the cornea, pricking in the pigment at every point, until it is completely covered. No inflammation follows this procedure, if it is skillfully done, and its results are marvelous in correcting deformities and the defects of vision above mentioned.

